# Potential Distribution of *Linepithema humile* (Hymenoptera: Formicidae) in South Korea: An Ensemble Species Distribution Modeling Approach

**DOI:** 10.1002/ece3.72976

**Published:** 2026-02-03

**Authors:** Hyeban Namgung, Hyojoong Kim

**Affiliations:** ^1^ Regenerative Organic Agriculture Division National Institute of Agricultural Sciences, RDA Wanju Republic of Korea; ^2^ Animal Systematics Lab., Department of Biological Science Kunsan National University Gunsan Republic of Korea

**Keywords:** BIOMOD2, ensemble modeling, extrapolated projection, invasive alien species, *Linepithema humile*

## Abstract

*Linepithema humile* is a well‐known invasive alien species that was first detected in 2019 at the cargo handling area of Busan Station in South Korea and has since established a population near the discovery site. Despite sufficient time for potential spread, no additional occurrence records have been reported outside the initial detection site. To predict its potential distribution across South Korea, we constructed species distribution models (SDMs) using occurrence data from its native range in South America and invasive range in North America. Modeling was conducted with the BIOMOD2 platform using five algorithms (ANN, GBM, MARS, MAXENT, and RF) and six environmental variables (Bio03, Bio04, Bio13, Bio16, Bio18, and the mean annual cumulative soil temperature degree‐days above 15.9°C). Model performance was evaluated with Kappa, ROC, and TSS, and only models meeting thresholds (Kappa ≥ 0.7, ROC ≥ 0.7, and TSS ≥ 0.5) were used for ensemble modeling via EMmean, EMwmean, and EMca methods. Projection accuracy was assessed using chi‐square tests based on occurrence data not used in model training. The predicted potential distribution included southern and southwestern coastal areas, which was consistent with the record in Busan. This study demonstrates the utility of SDMs trained on occurrence data from outside Korea in predicting the potential distribution of *L. humile* with limited domestic records and highlights high‐risk areas beyond Busan. Such approaches may support early detection and management strategies in the initial stages of biological invasion.

## Graphical abstract

The Argentine ant ( *Linepithema humile* ) was first discovered in Korea in Busan in 2019. Due to a lack of occurrence data, its distribution in Korea remains difficult to predict. This study constructed a single and ensemble model based on BIOMOD2 using occurrence data and environmental variables from South and North America and projected it to Korea. The predictions confirmed a high probability of distribution along the southern coast and Jeju, which can be used to develop countermeasures against the spread or further invasion of Argentine ants.

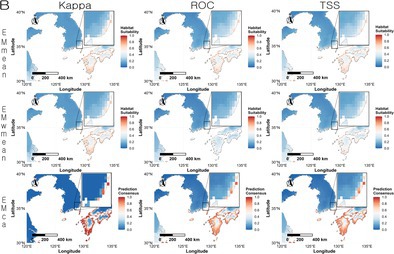

## 1 Introduction

Argentine ant, *Linepithema humile* (Mayr, 1868), is one of the most notorious ants known worldwide as an invasive alien species (Wild 2004; Luque et al. 2014). This species belongs to the subfamily Dolichoderinae (Hymenoptera: Formicidae). It is a small brownish ant with large eyes and a forward‐slanting propodeum, and it is also completely smooth and hairless on its pronotum and the front of its gaster (Wild 2007). This ant is native to the Paraná River basin in South America and is distributed in Argentina, Uruguay, Paraguay, and Brazil, as well as Chile, Colombia, Ecuador, and Peru (Wild 2004; Suarez et al. 2001). The species was probably introduced worldwide by trade in the 1800s, with the first confirmed invasion from Madeira Island in 1882 and later reported in many regions in the 1950s (Suarez et al. 2001; Tsutsui et al. 2001). It has now invaded six continents and is distributed in 78 regions in 36 countries (Gómez and Abril 2022).

They can establish nests in a variety of habitat types, including urban and human‐disturbed areas as well as in many natural environments, although these ants prefer subtropical and Mediterranean climates and their distribution is limited by cold, very dry, and humid conditions (Suarez et al. 2001; Espadaler and Gómez 2003; Krushelnycky et al. 2005; Brightwell et al. 2010). It has been reported that these invasive ants can compete with native ants in the invaded area, occupy the niche of native species (Human and Gordon 1996; Kennedy 1998; Carpintero et al. 2005; Touyama et al. 2003), thereby causing changes in the arthropod fauna and resulting in negative ecological effects (Cole et al. 1992; Bolger et al. 2000; de Mévergnies et al. 2024; Estany‐Tigerström et al. 2010). In addition, they can affect agriculture and sericulture through interactions with agricultural pests and virus transmission to honeybees (Sébastien et al. 2015; Yoo et al. 2013; Milosavljević et al. 2024; Daane et al. 2007; Powell and Silverman 2010). A total of approximately US$4 million has been spent worldwide on postinvasion management of this species (Angulo et al. 2022).

In South Korea, this species was first discovered in October 2019 on roads and container storage yards near Busan Station, and its establishment in the country was confirmed through monitoring (Lee et al. 2020; National Institute of Ecology 2020). Since then, the population has decreased through control measures, including high‐pressure insecticide spraying and the use of ant traps. However, no successful eradication has been reported to date (National Institute of Ecology 2020). The primary mode of dispersal for this species is accidental transport through human activities, which has facilitated its introduction to Korea and other parts of the world (Suarez et al. 2001). Now, in 2025, 5 years have passed since this species was first discovered in 2019. During this period, it may have spread to other regions of the country due to cargo and human movement, which could have a significant negative impact on the ecosystem in South Korea.

Species distribution modeling (SDM) is a useful tool for understanding the potential establishment and spread of invasive species (Barbet‐Massin et al. 2018; Miller 2010; Mainali et al. 2015). SDMs are constructed by quantifying the spatial correlation between species and geographical environment using statistical or machine learning methods, incorporating both species occurrence data and environmental data (Miller 2010; Elith and Leathwick 2009).

There are many advanced species distribution models, but the data requirements vary across models, and the results of modeling also vary depending on the model algorithm used, making it difficult to determine which model is appropriate (Dormann et al. 2018). In addition, models calibrated to existing data do not always guarantee good predictive power for new data independent of existing data (Araújo and New 2007). One approach to reducing uncertainty and discrepancies in model outcomes is to ensemble prediction, which integrates the predictive results of multiple models (Araújo and New 2007; Marmion et al. 2009; Thuiller et al. 2009).

Ensemble prediction aims to analyze the distribution of the entire ensemble results rather than selecting the model that best fits the observed data among several models to help reduce the influence of noise and uncertainty in the data and modeling, allowing us to capture the true signal we seek (Araújo and New 2007). There are several ways to use ensemble model predictions. A simple approach is to calculate the average or select the median of the predictions. More complex methods include Committee averaging, which first binarizes predictions before averaging, and the weighted mean, which assigns weights to predictions based on evaluation scores during model evaluation (Araújo and New 2007; Hao et al. 2019; Thuiller 2024).

Several studies have been conducted on species distribution modeling of Argentine ants. Roura‐Pascual et al. (2004) used species occurrence/absence data from South America, the native range of Argentine ants, and prepared two types of datasets for environmental variables: one including topographic and climate variables, and the other including topographic variables and normalized difference vegetation index (NDVI). Using the genetic algorithm for rule‐set production (GARP), a species distribution model, they predicted the potential distribution of Argentine ants in South America and globally. They also incorporated future climate scenario data to illustrate distribution changes between the present and 2050. Moriguchi et al. (2015) estimated the invasion and colonization risk map of Argentine ants in Japan by modeling seven environmental variables and 12 occurrence sites considered effective variables for ant species in WorldClim data using MAXENT. Jung et al. (2022) used CLIMEX to model globally and extract climatic preferences for major habitat locations using probability density functions. Li et al. (2022) predicted the potential habitat suitability of Argentine ants in China using MAXENT based on 2432 global occurrence records and 10 bioclimatic variables. The potential distribution of Argentine ants has been predicted, and the risk of invasion estimated from a global scale to some regions using a species distribution model, but there has been no analysis for Korea. It is obvious that the potential distribution in Korea should be assessed through species distribution modeling, which would facilitate the identification of monitoring areas and the development of additional control plans.

In this study, due to the lack of occurrence data on Argentine ants in South Korea, we modeled their native range in South America and the invaded region in North America, where sufficient occurrence data are available. We created both a single model and an ensemble model, which were then projected onto the Korean Peninsula to identify potential distribution of Argentine ants.

## 2 Materials and Methods

### 2.1 Modeling Platform

BIOMOD2, an R‐based ensemble platform for species distribution modeling, supports 11 types of species distribution models (Guéguen et al. 2025). It offers functions for generating and combining pseudo‐absence datasets, model calibration and evaluation, model ensembling, ensemble forecasting, and projection (Thuiller 2024). This tool is freely available and widely used by SDM researchers (Hao et al. 2019, 2020).

We built an ensemble of species distribution models using five algorithms in BIOMOD2: artificial neural networks (ANN), generalized boosting models (GBM), multiple adaptive regression splines (MARS), maximum entropy (MAXENT), and random forests (RF).

ANN is a nonlinear predictive model that imitates the structure of neural networks in the human brain (Lek, Delacoste, et al. 1996). It learns through hierarchical architecture consisting of an input layer, one or more hidden layers, and an output layer (Lek, Delacoste, et al. 1996; Venables and Ripley 2002). This model is used to predict species distributions by capturing complex relationships between environmental variables and species presence–absence data (Lek, Belaud, et al. 1996; Botella et al. 2018). GBM, also known as Boosted Regression Trees (BRT), is a model that combines regression trees and boosting algorithm (Friedman 2001). It trains several weak regression trees sequentially, and at each step, new trees are added to compensate for the residuals left by the previous trees, thereby improving the prediction performance (Friedman 2001). MARS is a regression‐based model that approximates complex nonlinear relationships between environmental variables and presence–absence data by constructing a sum of piecewise linear basis functions (Friedman 1991). MAXENT is a model that predicts species distribution through the maximum‐entropy principle. It predicts species distribution by finding a probability distribution that has maximum entropy while satisfying conditions that can restrict species distribution, that is, environmental variables and species occurrence data (Phillips et al. 2006). RF is an ensemble learning algorithm that randomly samples data using the bootstrap method, randomly selects a portion of the predictor variables for each tree to learn multiple decision trees and then performs the final prediction by voting (classification) or averaging (regression) the prediction results (Cutler et al. 2007).

### 2.2 Collection and Preparation of Presence and Pseudo‐Absence Data

Occurrence data of Argentine ants were obtained from the GBIF database, which is a worldwide occurrence data (Telenius 2011). The data was saved in the form of an xlsx file. In the occurrence data, the field values of the basis of record, such as living specimen and preserved specimen, were excluded from the coordinates that could not represent the habitat, and duplicate data and coordinates outside the range of environmental variables used in modeling were deleted. After that, the coordinates of the American continent were extracted, and the Spatially Rarefy Occurrence Data for SDMs Resolution to Rarefy Data of SDMtoolboxPRO in ArcGIS Pro were used to reduce spatial multicollinearity (Brown et al. 2017). The coordinates were set to 50 km for the resolution to rarefy data to prevent the coordinates from being clustered. Finally, 267 points were used to build and verify the species distribution model. The coordinate data other than the American continent was processed in the same way, and a total of 181 points were used to evaluate the accuracy of the projection results for the final ensemble model.

To accommodate models requiring absence data, we generated pseudo‐absence points using the BIOMOD_FormatingData() function in the biomod2 package. We created 801 points (three times the number of occurrence records) using the random strategy and repeated this process 10 times to produce 10 pseudo‐absence datasets (Figure S1).

### 2.3 Environmental Variable Data

We used bioclimatic variables for Worldclim's 1970–2000 historical climate data as environmental variable data (Fick and Hijmans 2017). These bioclimatic variables are widely used in species distribution modeling and are biological climate indices related to species distribution derived from temperature and precipitation data (Nix 1986; Hijmans et al. 2005; Booth et al. 2014). Worldclim provides 19 bioclimatic variables with a resolution of 10 arcminute (~340 km^2^), 5 arcmin (~86 km^2^), 2.5 arcmin (~21 km^2^), and 30 arcseconds (~1 km^2^). We selected the 5 arcmin bioclimatic variables, which have a spatial resolution closest to that of the cumulative degree‐days variable described below (Table S1).

We compiled biological traits of the Argentine ant from the literature (Table 1) to identify additional environmental predictors beyond bioclimatic variables that could constrain its occurrence as well as to select variables for modeling. Most studies on the ants' development and survival focused on temperature‐related factors, so we included cumulative soil temperature degree‐days above 15.9°C (Soildegree) which does not overlap with existing bioclimatic variables as an environmental variable.

**TABLE 1:** Biological information of *L. humile* in literature.

Biological parameters	Stage	Information	References
Lower development threshold (°C)	Egg	~18	Abril et al. (2010)
Upper development threshold (°C)	Egg	32	Abril et al. (2010)
Optimal development temperature (°C)	Egg	26	Abril et al. (2010)
Low lethal temperature (°C)	Adult (workers)	−10.5 to −4	Jumbam et al. (2008)
Upper lethal temperature (°C)	Adult (workers)	46	Holway et al. (2002)
	Adult (workers)	47	Walters and Mackay (2004)
	Adult (workers)	37 to 44	Jumbam et al. (2008)
Cumulative degree‐days above a 15.9°C	Egg to adult (workers)	445.4	Hartley and Lester (2003)
Activity temperature (°C)	Adult	5 to 35	Markin (1970)
Foraging temperature (°C)	Adult	10 to 30	Markin (1970)
Activity soil temperature (°C)	Adult	15 to 19	Witt and Giliomee (1999)
Foraging soil temperature (°C)	Adult	15 to 32	Hedges (1998)
Foraging stop soil temperature (°C)	Adult	40.8 to 44.8	Holway et al. (2002)
Habitat limiting factors	All stages	Dry climate	Ward (1987), Van Schagen et al. (1993), Kennedy (1998), Harris (2002)
	All stages	High rainfall environment	Vega and Rust (2001), Harris (2002)
Latitude of primary occurrence in the hemisphere	All stages	30 to 36	Harris (2002)
Distances spread of populations	Adult (queen)	150 m/year	Suarez et al. (2001)
Survival time of populations	All stages	14.1 years	Cooling et al. (2012)

To create the cumulative degree‐days variable, we downloaded the daily Soil Temperature Level 1 data on the ERA5‐Land hourly data from 1950 to present dataset from Copernicus Climate Change Service Climate Data Store (Muñoz Sabater 2019). We then extracted records for 2019–2023 and, for each day, averaged the 00:00 and 12:00 readings to obtain a daily mean temperature. Next, we calculated daily degree‐days by subtracting 15.9°C (setting negative results to zero) and summed these values for each year. Finally, we averaged five annual totals to produce the mean annual cumulative soil temperature degree‐days above 15.9°C (Soildegree). The daily Soil Temperature Level 1 data are restricted to a 0.1° × 0.1° resolution (approximately 6 arcminutes). Accordingly, the derived degree‐days variable was produced at the same resolution. To use the Soildegree variable developed in this study, all bioclimatic variables were resampled to match its spatial resolution (0.1° × 0.1°).

**FIGURE 1:**
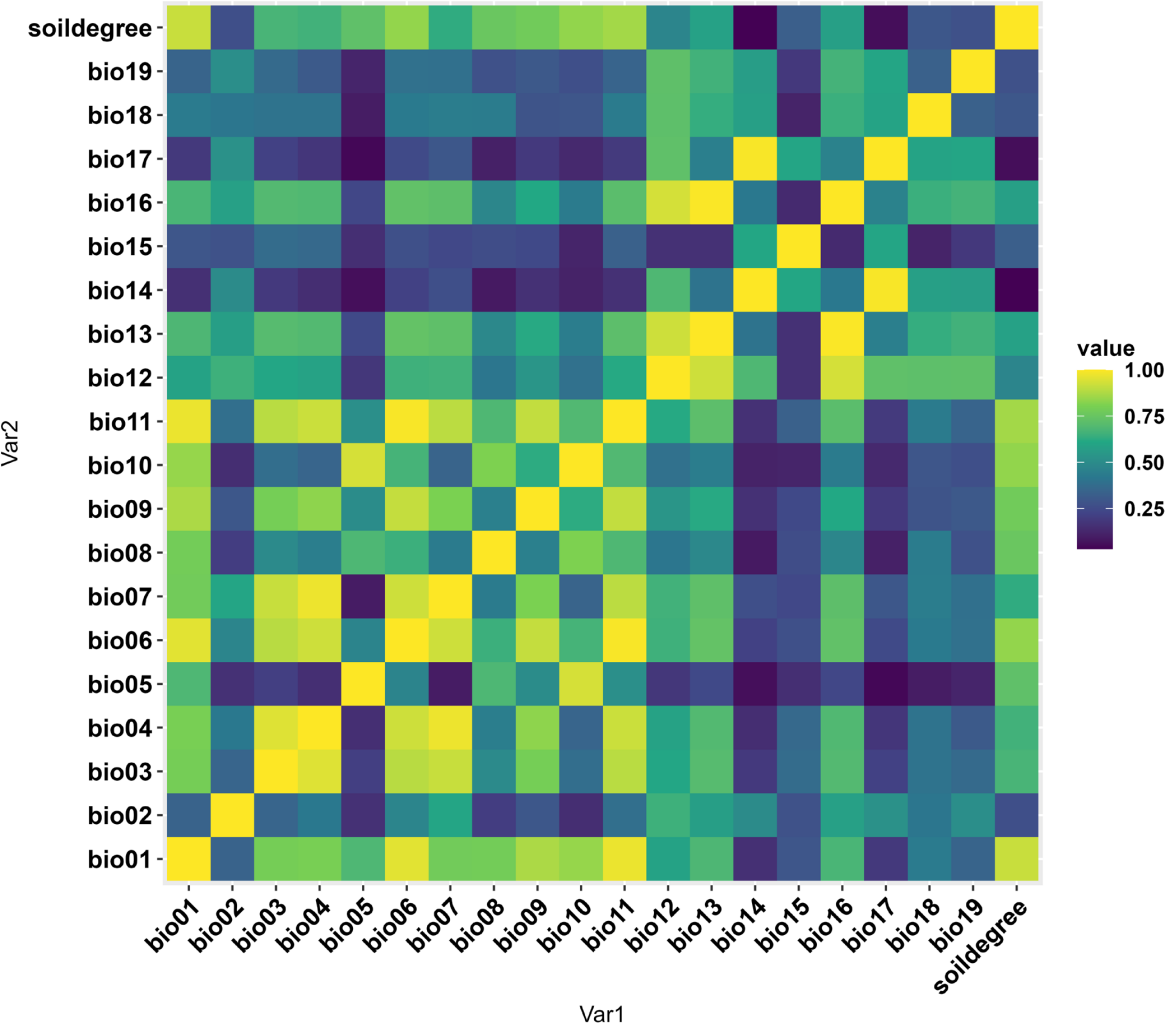
Pearson correlation heatmap of environmental variables. Colors represent the absolute values of Pearson's correlation coefficients (|*r*|), with yellow indicating stronger correlations and blue indicating weaker correlations. Correlation coefficients (|*r*|) range from 0 to 1.

All environmental variables were screened for pairwise Pearson correlation using the ENMTools package in R (Warren et al. 2021) (Figure 1). To avoid multicollinearity among highly correlated predictors, we constructed a variable set by excluding any pair of variables with an absolute correlation coefficient of 0.7 or higher (|*r*| < 0.7). We then selected and combined the remaining variables based on the biological characteristics of Argentine ants. There were initially six combinations of 11 variables, and after confirming the variable importance as a single modeling result for these combinations, we selected the top four variables among the average variable importance of all models and used them in the final modeling. As a result of confirming the variable importance of the six combinations, precipitation of warmest quarter (Bio18), and the mean annual cumulative soil temperature degree‐days above 15.9°C (Soildegree) were consistent in the top four, and isothermality (Bio03), temperature seasonality (Bio04), precipitation of wettest month (Bio13), and precipitation of wettest quarter (Bio16) were in the top four in the variable importance of the included combinations, respectively. The final variables selected were Bio03, Bio04, Bio13, Bio16, Bio18, and Soildegree. Modeling was conducted using three variable combination sets that accounted for correlations among these variables (Table 2).

**TABLE 2:** Summary of individual model performance and variable importance across three environmental variable sets.

Name	Variables	Models	Evaluation			Variable importance	
			Method	Number of model (≥ threshold)	Mean validation	Rank	Importance
Variable combination set 1	Bio03, Bio16, Bio18, Soildegree	ANN	Kappa	0	—	(1) Bio18	0.80
			ROC	117	0.76	(2) Soildegree	0.54
			TSS	20	0.56	(3) Bio16	0.43
						(4) Bio03	0.14
		GBM	Kappa	23	0.72	(1) Soildegree	0.37
			ROC	150	0.90	(2) Bio03	0.29
			TSS	150	0.65	(3) Bio18	0.22
						(4) Bio16	0.12
		MARS	Kappa	2	0.71	(1) Bio18	0.40
			ROC	149	0.88	(2) Soildegree	0.25
			TSS	137	0.59	(3) Bio16	0.22
						(4) Bio03	0.2
		MAXENT	Kappa	3	0.71	(1) Bio18	0.43
			ROC	150	0.86	(2) Soildegree	0.23
			TSS	135	0.59	(3) Bio03	0.22
						(4) Bio16	0.19
		RF	Kappa	24	0.71	(1) Bio03	0.41
			ROC	150	0.90	(2) Bio18	0.36
			TSS	150	0.64	(3) Soildegree	0.29
						(4) Bio16	0.13
Variable combination set 2	Bio04, Bio16, Bio18, Soildegree	ANN	Kappa	0	—	(1) Bio04	0.80
			ROC	150	0.85	(2) Soildegree	0.73
			TSS	147	0.63	(3) Bio16	0.23
						(4) Bio18	0.22
		GBM	Kappa	20	0.72	(1) Soildegree	0.36
			ROC	150	0.91	(2) Bio04	0.34
			TSS	150	0.66	(3) Bio18	0.19
						(4) Bio16	0.09
		MARS	Kappa	1	0.73	(1) Bio18	0.34
			ROC	150	0.88	(2) Bio04	0.25
			TSS	140	0.60	(3) Soildegree	0.25
						(4) Bio16	0.17
		MAXENT	Kappa	1	0.70	(1) Bio18	0.41
			ROC	150	0.87	(2) Bio04	0.27
			TSS	133	0.60	(3) Soildegree	0.21
						(4) Bio16	0.15
		RF	Kappa	14	0.72	(1) Bio04	0.46
			ROC	150	0.90	(2) Bio18	0.32
			TSS	148	0.64	(3) Soildegree	0.27
						(4) Bio16	0.13
Variable combination set 3	Bio04, Bio13 Bio18, Soildegree	ANN	Kappa	3	0.71	(1) Bio04	0.80
			ROC	148	0.85	(2) Soildegree	0.73
			TSS	140	063	(3) Bio18	0.21
						(4) Bio13	0.18
		GBM	Kappa	19	0.72	(1) Soildegree	0.37
			ROC	150	0.91	(2) Bio04	0.34
			TSS	150	0.66	(3) Bio18	0.20
						(4) Bio13	0.09
		MARS	Kappa	5	0.71	(1) Bio18	0.35
			ROC	150	0.88	(2) Soildegree	0.26
			TSS	142	0.60	(3) Bio04	0.25
						(4) Bio13	0.17
		MAXENT	Kappa	1	0.72	(1) Bio18	0.42
			ROC	150	0.86	(2) Bio04	0.27
			TSS	129	0.60	(3) Soildegree	0.22
						(4) Bio13	0.14
		RF	Kappa	15	0.72	(1) Bio04	0.46
			ROC	150	0.90	(2) Bio18	0.32
			TSS	146	0.63	(3) Soildegree	0.27
						(4) Bio13	0.13

*Note:* Model performance was evaluated using Kappa, ROC (AUC), and TSS. Number of model (≥ threshold) indicates the number of model runs meeting the evaluation criteria (Kappa ≥ 0.7, ROC ≥ 0.7, and TSS ≥ 0.5). Environmental variables are defined as follows: Bio03, isothermality; Bio04, temperature seasonality; Bio13, precipitation of the wettest month; Bio16, precipitation of the wettest quarter; Bio18, precipitation of the warmest quarter; and Soildegree, mean annual cumulative soil temperature degree‐days above 15.9°C.

### 2.4 Modeling Procedure

Single modeling was performed using five algorithms across three different combinations of environmental variables. Each modeling was performed using the BIOMOD_Modeling() function in the biomod2 package, and the parameters of each model were set according to the predefined bigboss configuration provided by the biomod2 team. In biomod2, variable importance is assessed using the permutation importance method, which evaluates the relative contribution of each variable. It is calculated as 1 minus the Pearson correlation coefficient between the predictions of the original model and those of a model in which the values of a single variable have been randomly shuffled. The number of permutations used to assess variable importance was set to 5. Cross‐validation was performed using 5‐fold cross‐validation repeated three times, and model performance was evaluated using Cohen's κ (Kappa), the True Skill Statistic (TSS), and the Receiver Operating Characteristic curve (ROC). These three metrics are commonly used to evaluate species distribution models (Allouche et al. 2006; Leroy et al. 2018).

Kappa is used to evaluate the accuracy of presence–absence predictions in species distribution modeling (Allouche et al. 2006; McPherson et al. 2004; Segurado and Araújo 2004). It measures the level of agreement between the model's predictions and the observed data, while accounting for the agreement that could occur by chance. The Kappa value ranges from −1 to 1, where 1 indicates perfect agreement and values below 0 suggest performance no better than or worse than random prediction (Cohen 1960).

ROC is commonly used to evaluate the performance of models that produce continuous outputs, such as habitat suitability scores or predicted probabilities of presence (Fielding and Bell 1997; Lobo et al. 2008; Shabani et al. 2018). It is constructed by applying all possible thresholds to convert continuous predictions into binary presence–absence outcomes. For each threshold, a confusion matrix is generated to compute sensitivity and specificity. These values are then used to plot true positive rate (sensitivity) against the false positive rate (1 − specificity) (Fielding and Bell 1997; Lobo et al. 2008; Shabani et al. 2018; Jiménez‐Valverde 2012). The model's performance is summarized using the AUC. AUC values range from 0 to 1, with a value of 0.5 indicating that the model's ability to discriminate between the presence and absence is equivalent to random chance and a value of 1 indicating perfect discriminatory ability (Fielding and Bell 1997; Jiménez‐Valverde 2012).

TSS is another metric used to evaluate the accuracy of presence–absence predictions, similar to the methods described above (Allouche et al. 2006). It is calculated by summing sensitivity and specificity, which are derived from a confusion matrix, and then subtracting one from the result (Allouche et al. 2006; Leroy et al. 2018; Somodi et al. 2017). These values range from −1 to 1, similar to Kappa, where 1 indicates perfect predictive performance, and values below 0 indicate performance worse than random prediction (Allouche et al. 2006).

We used the three evaluation metrics to select models for ensemble modeling based on each evaluation criterion. Only models with performance values equal to or above the defined thresholds were used in the ensemble modeling: Kappa ≥ 0.7 (Landis and Koch 1977; Monserud and Leemans 1992; Duan et al. 2014), ROC ≥ 0.7 (Duan et al. 2014; Swets 1988; Peterson et al. 2011), and TSS ≥ 0.5 (Shabani et al. 2018).

Ensemble modeling was performed using the BIOMOD_EnsembleModeling() function in the biomod2 package. The ensemble of single models was calculated based on the predefined ensemble algorithm and we selected the EMmean, EMca, and EMwmean algorithms to obtain the ensemble model outputs. EMmean is one of the simplest ensemble methods, which calculates the average suitability across selected models. EMca converts model outputs into binary data based on thresholds defined during single modeling, selecting values that maximize evaluation metrics on the test dataset. When ensemble predictions using the EMca approach 0 or 1, it indicates that all contributing models consistently predict absence (0) or presence (Wild 2004), respectively. EMwmean generates a weighted average of model predictions, where weights are assigned according to evaluation scores from individual models, and a higher weight is given to the model with a higher evaluation score. Each model ensemble of these three algorithms was projected to the global scale. The ensemble models generated using the EMmean and EMwmean algorithms were used to predict habitat suitability and subsequently evaluated through a chi‐square test. The ensemble model of EMca was used to check the binarized suitability prediction ratio of the models used in the ensemble.

To evaluate whether the spatial distribution of suitable areas predicted by the globally projected ensemble models differed from random expectations, we conducted chi‐square tests using occurrence data which were not included in modeling (i.e., data from outside the Americas) (Roura‐Pascual et al. 2004) (Figure S2). The model predictions were binarized using the average cutoff values of the individual models included in the ensemble (Table 3). Based on the proportion of the predicted presence area relative to the total land area, we calculated the expected number of test occurrences under a random distribution assumption. A chi‐square test with 1 degree of freedom was used to determine whether the observed number of occurrences in the predicted presence area significantly differed from the expected value. The chi‐square test was applied as a complementary, distribution‐level assessment rather than as a direct measure of predictive accuracy. The chi‐square test was analyzed using the chisq.test() function in R.

**TABLE 3:** Summary of the number of models above threshold and mean cutoff value for each set of variable sets.

Set	Evaluation method	Number of model (≥ threshold)	Mean of cutoff
Variable combination set 1	Kappa	52	0.520
	ROC	716	0.448
	TSS	592	0.440
Variable combination set 2	Kappa	36	0.536
	ROC	750	0.452
	TSS	718	0.454
Variable combination set 3	Kappa	43	0.545
	ROC	748	0.448
	TSS	707	0.450

*Note:* Number of models (≥ threshold) indicates the number of model runs meeting the evaluation criteria (Kappa ≥ 0.7, ROC ≥ 0.7, and TSS ≥ 0.5). The cutoff value represents the mean suitability threshold used to convert continuous habitat suitability outputs into binary predictions.

## 3 Results

### 3.1 Performance Evaluation of Single Species Distribution Models and Variable Importance According to Environmental Variable Combinations

**FIGURE 2:**
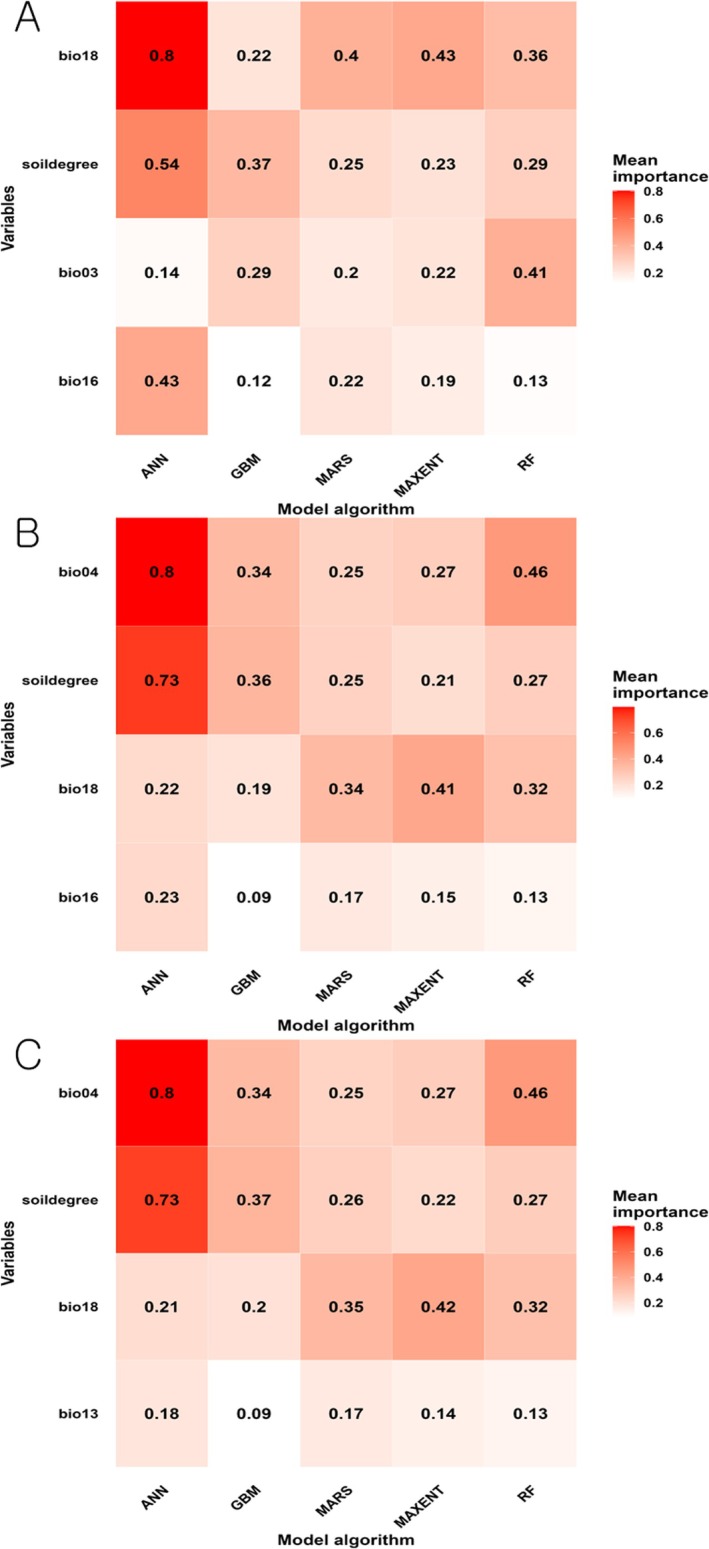
Heatmaps of variable importance across modeling algorithms for each variable combination set. (A) Variable combination set 1 (Bio03, Bio16, Bio18, and Soildegree); (B) variable combination set 2 (Bio04, Bio16, Bio18, and Soildegree); (C) variable combination set 3 (Bio04, Bio13, Bio18, and Soildegree). Colors indicate mean variable importance, with darker shades representing higher importance. Environmental variables are defined as follows: Bio03, isothermality; Bio04, temperature seasonality; Bio13, precipitation of the wettest month; Bio16, precipitation of the wettest quarter; Bio18, precipitation of the warmest quarter; and Soildegree, mean annual cumulative soil temperature degree‐days above 15.9°C.

A total of 150 models were generated for each modeling algorithm across the three variable combination sets. Each model was evaluated using Kappa, ROC, and TSS, which determined the number of models used in ensemble modeling and their average evaluation metrics. Across all algorithms and variable sets, models generally showed high performance for ROC and TSS, while Kappa yielded more conservative results, often failing to meet the threshold for certain algorithms like ANN (Table 2). While the relative contribution of each predictor varied across algorithms, variable importance showed consistent trends within each variable combination set (Figure 2).

In variable combination set 1 (Bio03, Bio16, Bio18, and Soildegree), precipitation of the warmest quarter (Bio18) and the mean annual cumulative soil temperature degree‐days above 15.9°C (Soildegree) were the most important predictors in most models, including ANN, MARS, and MAXENT. In contrast, the RF model showed a higher importance for isothermality (Bio03).

In variable combination set 2 (Bio04, Bio16, Bio18, and Soildegree), temperature seasonality (Bio04) was a dominant predictor, particularly in ANN and RF models. However, precipitation of the warmest quarter (Bio18) remained the primary predictor for MARS and MAXENT models.

In variable combination set 3 (Bio04, Bio13, Bio18, and Soildegree), temperature seasonality (Bio04) and Soildegree showed high importance across models. Precipitation of the warmest quarter (Bio18) also consistently showed high importance across all algorithms, whereas precipitation of the wettest month (Bio13) had a relatively lower impact than the other predictors.

### 3.2 The Response Curves of the Variables Were Visualized for Each Single Model

**FIGURE 3:**
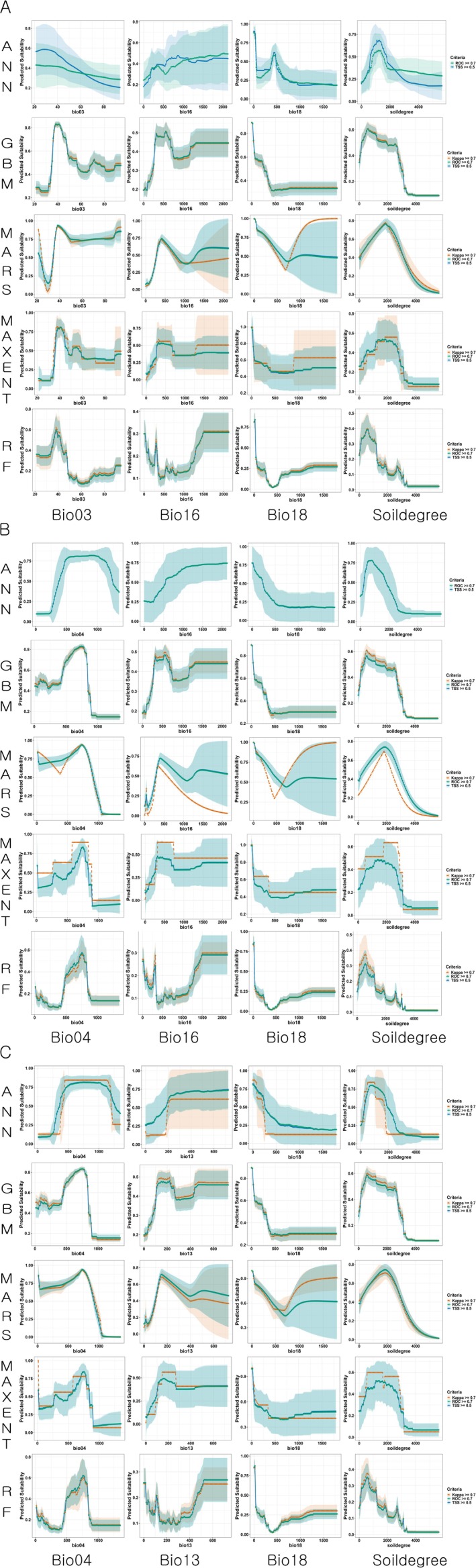
Response curves of environmental variables for each algorithm used in the ensemble modeling. Orange triangles indicate response curves from model runs with Kappa ≥ 0.7, green squares indicate those with ROC ≥ 0.7 and blue circles represent those with TSS ≥ 0.5. A, variable combination set 1 (Bio03, Bio16, Bio18, and Soildegree); B, variable combination set 2 (Bio04, Bio16, Bio18, and Soildegree); C, variable combination set 3 (Bio04, Bio13, Bio18, and Soildegree).

Each response curve shows the mean variable response calculated from the models that met the threshold criteria for evaluation metrics and were selected for ensemble modeling (Figure 3).

In the variable combination set 1, the modeling was conducted using isothermality (Bio03), precipitation of the wettest quarter (Bio16), precipitation of the warmest quarter (Bio18), and the mean annual cumulative soil temperature degree‐days above 15.9°C (Soildegree) as modeling variables. In most models, the habitat suitability decreased as the precipitation of the warmest quarter (Bio18) increased. The mean annual cumulative soil temperature degree‐days above 15.9°C (Soildegree) showed a high habitat suitability in the range of approximately 1000–2000. For isothermality (Bio03), the ANN and RF models exhibited a decreasing trend in suitability as the value increased, whereas the other three models showed an initial increase, and then a slight decrease. precipitation of the wettest quarter (Bio16) showed a tendency to increase the habitat suitability as its value increased, despite some model‐specific variability (Figure 3A).

In variable combination set 2, the modeling was conducted using temperature seasonality (Bio04), precipitation of wettest quarter (Bio16), precipitation of warmest quarter (Bio18), and the mean annual cumulative soil temperature degree‐days above 15.9°C (Soildegree). temperature seasonality (Bio04) showed a high suitability in the range of 500–1000 in most models. The response curves of precipitation of wettest quarter (Bio16), precipitation of warmest quarter (Bio18), and the mean annual cumulative soil temperature degree‐days above 15.9°C (Soildegree) showed similar patterns to those observed in variable combination set 1 (Figure 3B).

In variable combination set 3, the modeling was conducted using temperature seasonality (Bio04), precipitation of wettest month (Bio13), precipitation of warmest quarter (Bio18), and the mean annual cumulative soil temperature degree‐days above 15.9°C (Soildegree). The response curve of precipitation of wettest month (Bio13) was similar to that of precipitation of wettest quarter (Bio16) in variable combination set 2, and the other variables also exhibited response patterns similar to those in variable combination set 2 (Figure 3C).

### 3.3 Model Selection for Ensemble Modeling and Cutoff for Binarization Map

The models for each variable combination were selected for the ensemble based on the threshold of each evaluation metric (Table 3).

In variables combination set 1 (Bio03, Bio16, Bio18, and Soildegree), 52 models met the thresholds of Kappa ≥ 0.7, 716 models met ROC ≥ 0.7, and 592 models met TSS ≥ 0.5. The corresponding mean cutoff values were 0.520 for Kappa, 0.448 for ROC, and 0.440 for TSS.

In variable combination set 2 (Bio04, Bio16, Bio18, and Soildegree), 36 models satisfied the Kappa threshold, while 750 and 718 models met the ROC and TSS thresholds, respectively. The corresponding mean cutoff values were 0.536 for Kappa, 0.452 for ROC, and 0.454 for TSS.

In variable combination set 3 (Bio04, Bio13, Bio18, and Soildegree), 43 models satisfied the Kappa threshold, 748 models met the ROC threshold, and 707 models met the TSS threshold. The corresponding mean cutoff values were 0.545 for Kappa, 0.448 for ROC, and 0.450 for TSS.

These mean cutoff values were used as thresholds to generate binary maps representing the predicted presence and absence of *L. humile* based on global projections of the ensemble models. The resulting binary maps were also used in chi‐square tests to evaluate the predictive performance of the ensemble models.

### 3.4 Projection Results of the Ensemble Model

**FIGURE 4:**
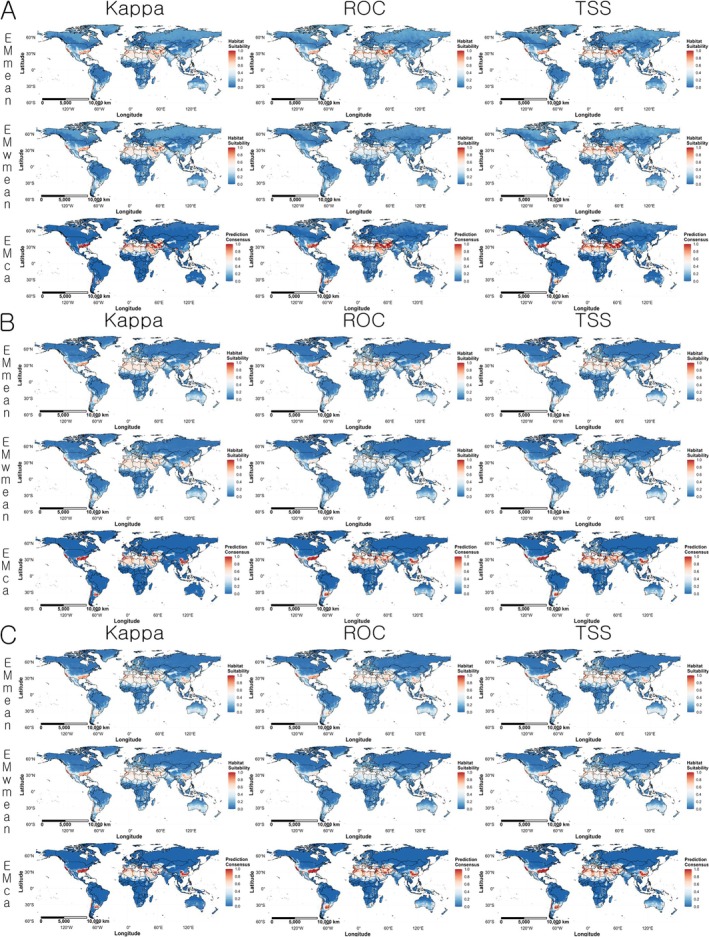
Global prediction maps of *Linepithema humile* habitat suitability based on ensemble models. Red indicates higher suitability, and blue indicates lower suitability. (A) Variable combination set 1 (Bio03, Bio16, Bio18, and Soildegree); (B) variable combination set 2 (Bio04, Bio16, Bio18, and Soildegree); (C) variable combination set 3 (Bio04, Bio13, Bio18, and Soildegree).

The global projections showed that highly suitable areas were mainly distributed between 20° and 40° north latitude regions. The main high habitat suitability areas were the eastern and western United States, central and southern Chile, parts of Argentina, the Mediterranean coast, North Africa, parts of East Africa, south of the Himalayas, eastern China, Taiwan, western and southern Japan, North Africa, and parts of East Africa (Figure 4).

**FIGURE 5:**
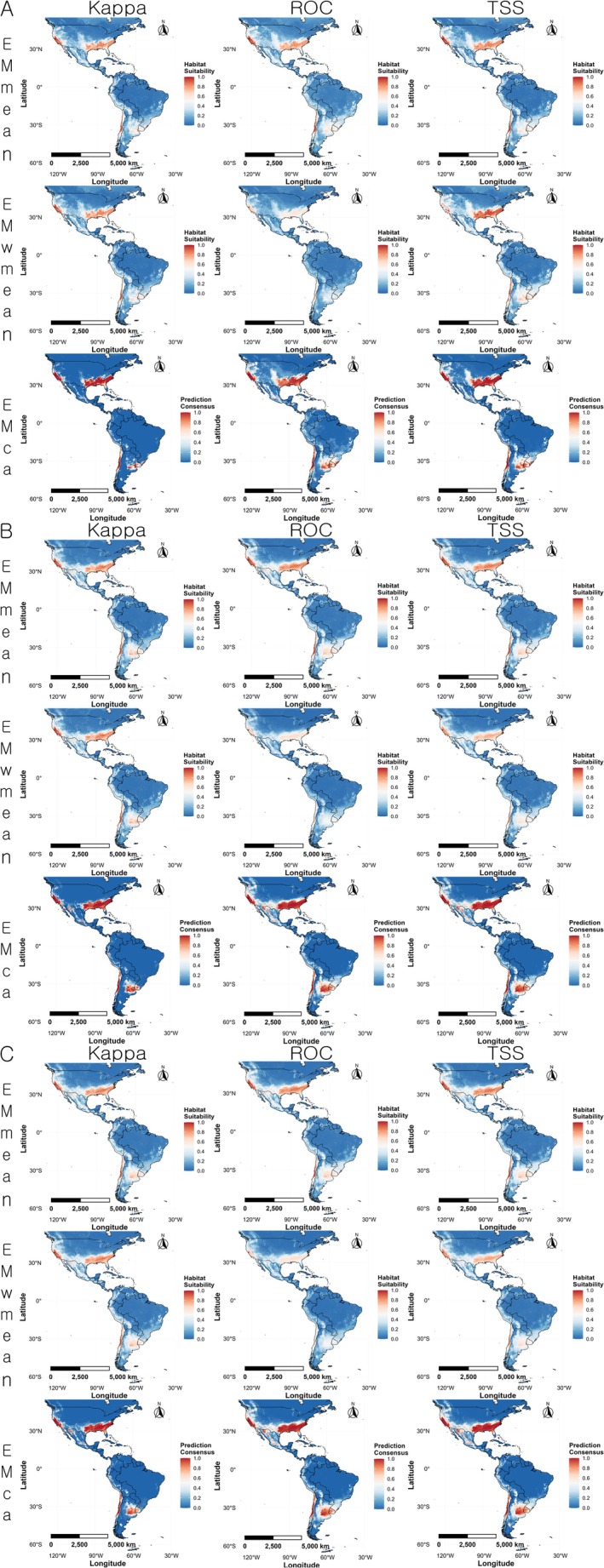
Regional prediction maps of *Linepithema humile* habitat suitability in the Americas based on ensemble models. Red indicates higher suitability, and blue indicates lower suitability. (A) variable combination set 1 (Bio03, Bio16, Bio18, and Soildegree); (B) variable combination set 2 (Bio04, Bio16, Bio18, and Soildegree); (C) variable combination set 3 (Bio04, Bio13, Bio18, and Soildegree).

In the Americas, the ensemble models from all three variable combination sets showed a strong agreement between areas with high suitability and actual occurrence locations. High suitability areas included the northeastern and northwestern United States, the Mexican highlands, parts of Central America, central and southern Chile, and the native range around the Paraná River basin. Compared to EMmean, the EMwmean projection showed broader areas with high habitat suitability. There were no significant differences between the ensemble results of variable combination set 2 and set 3 (Figure 5).

**FIGURE 6:**
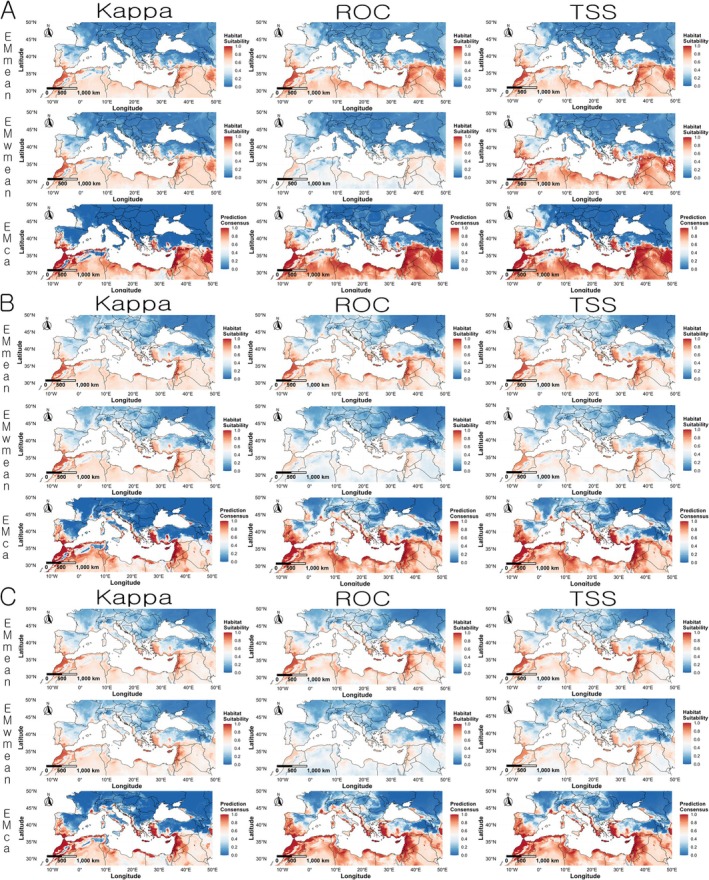
Regional prediction maps of *Linepithema humile* habitat suitability in Europe based on ensemble models. Red indicates higher suitability, and blue indicates lower suitability. (A) Variable combination set 1 (Bio03, Bio16, Bio18, and Soildegree); (B) variable combination set 2 (Bio04, Bio16, Bio18, and Soildegree); (C) variable combination set 3 (Bio04, Bio13, Bio18, and Soildegree).

In the Europe, all three variable combination sets predicted high habitat suitability along the Mediterranean coast, particularly in southern Spain, southern Italy, Greece, western Turkey, and parts of the Balkan Peninsula. When compared with the actual occurrence points, the prediction results matched most known presence locations in Europe, except for eastern Spain (Figure 6).

**FIGURE 7:**
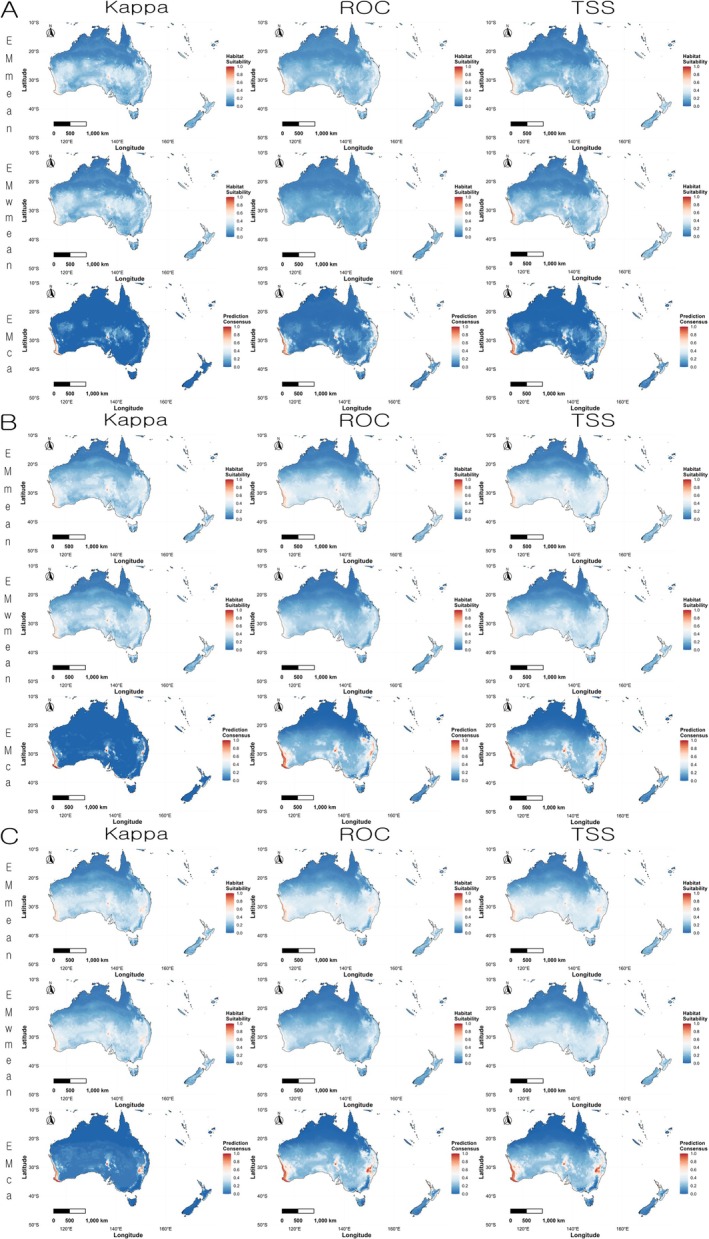
Regional prediction maps of *Linepithema humile* habitat suitability in Australia and New Zealand based on ensemble models. Red indicates higher suitability, and blue indicates lower suitability. (A) Variable combination set 1 (Bio03, Bio16, Bio18, and Soildegree); (B) variable combination set 2 (Bio04, Bio16, Bio18, and Soildegree); (C) variable combination set 3 (Bio04, Bio13, Bio18, and Soildegree).

In Australia and New Zealand, all three variable combination sets predicted high habitat suitability along the southwestern coast of Western Australia, the southeastern coast, parts of eastern Australia, and coastal areas of New Zealand's North Island. When comparing the actual occurrence points with the predicted results, the southwestern coast of Western Australia and the North Island of New Zealand showed a high degree of agreement, while other regions showed lower consensus (Figure 7).

**FIGURE 8:**
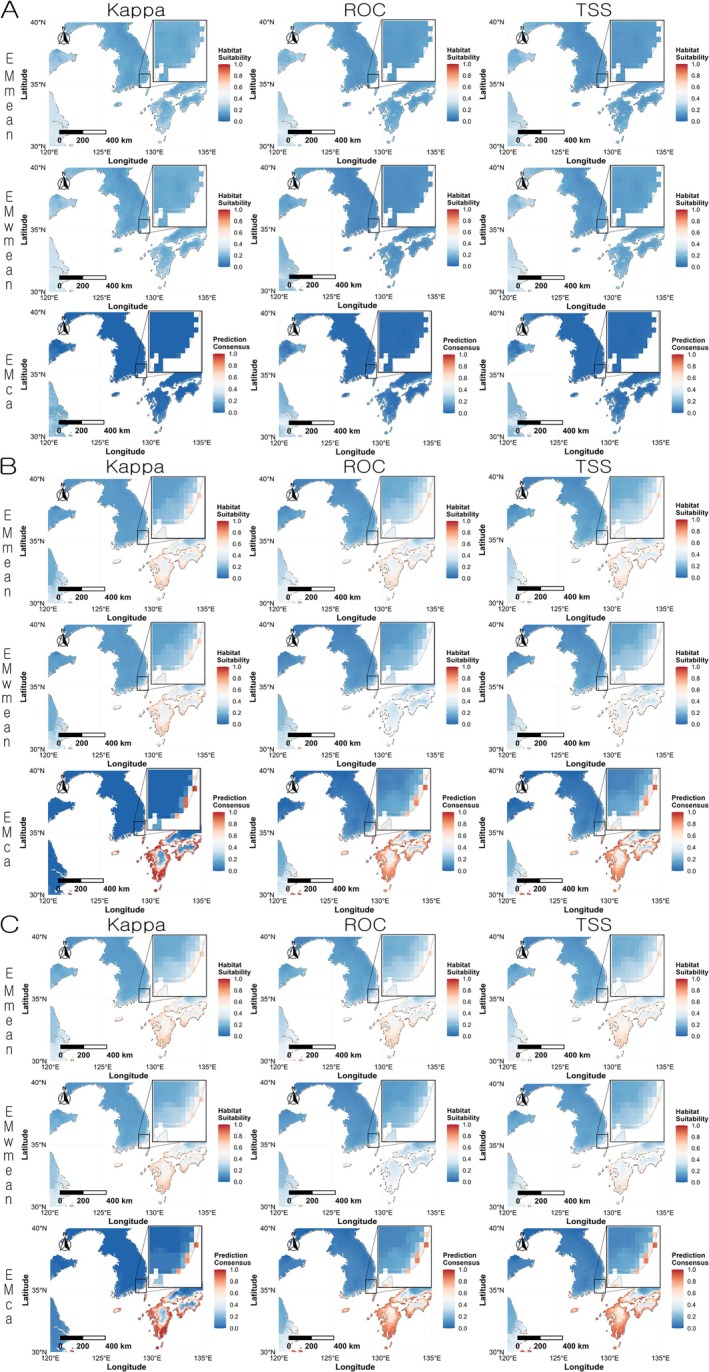
Regional prediction maps of *Linepithema humile* habitat suitability in South Korea based on ensemble models. Red indicates higher suitability, and blue indicates lower suitability. (A) Variable combination set 1 (Bio03, Bio16, Bio18, and Soildegree); (B) variable combination set 2 (Bio04, Bio16, Bio18, and Soildegree); (C) variable combination set 3 (Bio04, Bio13, Bio18, and Soildegree).

In the projection centered on the Korean Peninsula, variable combination set 2 and set 3 predicted a high occurrence probability in the southern part of the Korean Peninsula and the western and southern coasts of Japan. In contrast, variable combination set 1 predicted low habitat suitability across most areas in all ensemble algorithms. When compared with the actual occurrence points, *L. humile* has established around Busan Station in South Korea, and this was consistent with the predictions from variable combination set 2 and set 3 (Figure 8).

### 3.5 Chi‐Square Test Results of Ensemble Model Prediction Performance

To evaluate the prediction accuracy of the model, a chi‐square test was performed using 181 test data that were not used for learning (Table 4).

**TABLE 4:** Chi‐square test results comparing observed and predicted presence from ensemble model outputs for each variable combination set.

Set	Ensemble method	Evaluation method	No. test data point	Observed presence	Observed presence ratio	Predicted presence ratio	Expected presence	*χ* ^2^	*p*
Variable combination set 1	EMmean	Kappa	181	51	0.282	0.109	19.746	55.525	< 0.001
		ROC	181	78	0.431	0.135	24.403	136.059	< 0.001
		TSS	181	82	0.453	0.127	23.035	172.944	< 0.001
	EMwmean	Kappa	181	51	0.282	0.110	19.836	54.990	< 0.001
		ROC	181	45	0.249	0.062	11.146	109.577	< 0.001
		TSS	181	93	0.514	0.150	27.179	187.568	< 0.001
Variablecombination set 2	EMmean	Kappa	181	65	0.359	0.132	23.944	81.128	< 0.001
		ROC	181	111	0.613	0.140	25.419	335.214	< 0.001
		TSS	181	111	0.613	0.140	25.388	335.790	< 0.001
	EMwmean	Kappa	181	65	0.359	0.132	23.949	81.096	< 0.001
		ROC	181	60	0.331	0.052	9.377	288.227	< 0.001
		TSS	181	82	0.453	0.103	18.634	240.211	< 0.001
Variable combination set 3	EMmean	Kappa	181	60	0.331	0.083	14.987	147.407	< 0.001
		ROC	181	112	0.619	0.140	25.307	345.244	< 0.001
		TSS	181	112	0.619	0.139	25.149	348.334	< 0.001
	EMwmean	Kappa	181	60	0.331	0.081	14.732	151.424	< 0.001
		ROC	181	58	0.320	0.052	9.467	262.554	< 0.001
		TSS	181	92	0.508	0.110	19.953	292.381	< 0.001

*Note:* Two ensemble approaches were used: EMmean (mean ensemble of models) and EMwmean (weighted mean ensemble of models). Observed presence ratio is the proportion of test data points with observed presence and predicted presence ratio is the proportion of sites classified as presence based on the ensemble cutoff. Expected presence is the number of presences expected under the predicted ratio. The *χ* ^2^ values and *p*‐values indicate the deviation between observed and expected presences.

In variable combination set 1, the observed presence ratio was relatively low, ranging from 0.282 to 0.514, compared to set 2 and set 3. Most ensemble models predicted even lower presence ratios. Among them, the TSS‐based EMwmean model showed the highest observed presence ratio of 0.514 and yielded the highest chi‐square value. The model with the lowest predictive performance was the Kappa‐based EMwmean ensemble model, which had an observed presence ratio of 0.282 and a chi‐square value of 54.990. The Kappa‐based EMmean ensemble model also showed similar results.

In variable combination set 2, the observed presence ratio ranged from 0.359 to 0.613. The ROC‐ and TSS‐based EMmean ensemble models both showed high observed presence ratios of 0.613, along with high chi‐square values of 335.214 and 335.790, respectively. The lowest observed presence ratio was found in the ROC‐based EMwmean ensemble model (0.331), but it still yielded a chi‐square value of 288.227, which was higher than those of the Kappa‐based ensemble models.

In variable combination set 3, a similar trend to set 2 was observed. The ROC and TSS‐based models showed the highest observed presence ratio. In particular, the ROC‐ and TSS‐based EMmean ensemble models showed the highest observed presence ratio of 0.619 and the highest chi‐square values of 345.244 and 348.334, respectively, and these models showed the highest performance across all models.

Overall, model predictions were statistically significantly different from random expectations across all three variable combination sets and ensemble methods (*p* < 0.001), indicating the predicted spatial distribution of the ensemble model is significantly more consistent with the observed occurrence pattern than expected under a random distribution. In particular, the ROC‐ and TSS‐based EMmean ensemble models in Set 2 and Set 3 showed the highest prediction accuracies and clear prediction trends.

### 3.6 Overall Performance of Ensemble Models

We conducted modeling for each of the three environmental variable combination sets, selected models based on three evaluation metrics (Kappa, ROC, and TSS), and then created ensemble models using three ensemble algorithms to predict the global habitat suitability of the Argentine ant.

In all three combination sets of variables, models based on variable combination set 1 resulted in fewer models eligible for ensemble modeling compared to set 2 and set 3. This set also showed the lowest chi‐square statistics and a relatively low observed presence ratio. Although set 3 yielded slightly higher chi‐square values and observed presence ratios than set 2, the differences were not substantial.

Among the evaluation metrics, Kappa yielded the fewest models suitable for ensemble modeling compared to ROC and TSS. In some algorithms, the number of usable models under the Kappa criterion was as low as zero or one. For ROC and TSS, more than 120 models were eligible for ensemble modeling in all variable sets except set 1. Ensemble models based on ROC and TSS also exhibited higher chi‐square values and observed presence ratios than those based on Kappa. In terms of spatial projections, the Kappa‐based ensemble predicted a more conservative distribution range, while the ROC‐based ensemble tended to produce relatively weaker habitat suitability compared to Kappa and TSS.

## 4 Discussion

### 4.1 Extrapolation and Model Reliability

Our goal is to explore the potential distribution of Argentine ants across the Korean Peninsula beyond the currently known occurrence at Busan Station. However, the distribution of Argentine ants in South Korea is currently limited to a single locality, making it difficult to predict potential distribution across the Korean Peninsula using interpolation. Therefore, we address this issue by applying an extrapolation‐based ecological niche modeling approach. We selected the Americas as a training area, as South America is the native range of the Argentine ant and its relatively long invasion history in the Americas has provided sufficient distribution records (Suarez et al. 2001; Sanders et al. 2001). Moreover, the distribution of this species across a broad latitudinal gradient enables the incorporation of its responses to a wide range of climatic conditions into the model, avoiding sampling biases often associated with global records (Hughes et al. 2021; Phillips et al. 2009).

Although predictions based on extrapolation can be inherently uncertain (Heikkinen et al. 2012; Guillaumot et al. 2020), our examination using Multivariate Environmental Similarity Surfaces (MESS) indicated that South Korea was predicted under interpolated environmental conditions rather than extrapolated ones (Figure S3). This outcome suggests that the broad geographic and latitudinal extent of the Americas includes climatic zones comparable to those found in the Korean Peninsula, thereby supporting the reliability of our projections (Guillaumot et al. 2020; Elith et al. 2010).

### 4.2 Environmental Factors Associated With Distribution

Among the environmental variables, the mean annual cumulative soil temperature degree‐days above 15.9°C (Soildegree) was identified as the most important predictor in most models. Response curve analyses showed a high habitat suitability within the range of 500–2000 degree‐days, which covers approximately 95.8% of South Korea's territory (Figure S4). This indicates that thermal accumulation for worker development is sufficient across most of the country. When converted to annual mean temperature, this range corresponds approximately to 17.3°C–21.3°C, which corresponds to both the native range and other known invaded areas (Jung et al. 2022; Harris 2002). According to Hartley and Lester (Hartley and Lester 2003), the developmental threshold for workers is 445 degree‐days above 15.9°C, which closely matches the observed response curve. Most occurrence records in Argentina ant also fall within this temperature accumulation range. Notably, the habitat suitability declined sharply when degree‐days exceeded 2000, suggesting that extremely hot regions may be unsuitable for survival (Abril et al. 2010).

The next most important variable was temperature seasonality (Bio04). The response curve indicated a high habitat suitability between values of approximately 500 and 800, which corresponds to regions with relatively low seasonality. The native range of the Argentine ant is located in a humid subtropical climate with relatively mild winters. This species is biologically known to prefer temperatures around 30°C. Most recorded occurrences are found in areas where the standard deviation of temperature seasonality (Bio04) is between 5°C and 8°C, suggesting that Argentine ants prefer regions without extreme seasonal temperature differences. While thermal accumulation is widespread, this optimal seasonality range accounts for only 2.01% of South Korea's territory (Figure S5). In South Korea, these conditions are mainly found in southern coastal areas and Jeju Island.

Although extremely wet environments can limit the distribution of Argentine ants (Harris 2002; Vega and Rust 2001), overly dry climates can also act as a constraint. However, Argentine ants have been observed to persist in regions, such as California, Portugal, and Spain, where precipitation remains low during the warm season (Jung et al. 2022). Areas with high isothermality (Bio03) values were mainly located near the equator, which may be due to the negative impact of tropical climates and high temperature variability on Argentine ant occurrence. This pattern is consistent with previous studies that equatorial or highly variable thermal environments can suppress ant activity or seasonality (Parr and Bishop 2022; Tozetto et al. 2023).

### 4.3 Comparison With Previous Studies

When comparing our results with previous studies on the species distribution modeling of Argentine ants, Roura‐Pascual et al. (Roura‐Pascual et al. 2004) conducted a global‐scale SDM using the Genetic Algorithm for Rule‐set Prediction (GARP) model, incorporating species occurrence data, climate variables, topographic data, and the Normalized Difference Vegetation Index (NDVI). However, the predicted distribution in their study differed substantially from ours, especially in South Korea. While Roura‐Pascual et al. suggested potential distribution across the entire Korean Peninsula or concentrated in its western regions, our results indicated a high habitat suitability specifically along the southern coastal areas.

In the Iberian Peninsula, Roura‐Pascual et al. (2009) performed an ensemble prediction of potential distribution areas in the Iberian Peninsula using five different modeling algorithms. When compared with our prediction results, their model showed a similar distribution pattern except in the eastern part of the Iberian Peninsula, despite differences in environmental variables and the modeling algorithms.

The habitat suitability results from the CLIMEX model developed by Jung et al. (2022) showed a similar spatial pattern to our predicted habitat suitability, although the extent differed. Jung et al. predicted high habitat suitability extending from southern to central Africa and from eastern Brazil to the Paraná River drainage basin, whereas our results indicated only moderate habitat suitability in limited parts of southern Africa and high suitability observed in the Paraná River basin and central Chile. Our predictions for the Middle East and African desert regions indicated high habitat suitability, but these areas fall largely outside the range of the environmental conditions used to train the models. Therefore, the reliability of these results is low, as they are heavily influenced by extrapolation (Guillaumot et al. 2020; Elith et al. 2010).

The ensemble modeling approach reduces variance between models and resolves differences between algorithms. This provides more stable estimates of the distribution area for management purposes (Harris et al. 2024; Ramirez‐Reyes et al. 2021). The projections for the Korean Peninsula in this study contribute to more reliable identification of high‐risk areas for Argentine ants. These results provide a practical basis for developing strategies for monitoring and early management of *L. humile* .

### 4.4 Predicted High‐Risk Areas for Monitoring in South Korea

In South Korea, excluding Busan Station, the areas predicted to have a high habitat suitability were Ulsan, Geoje, Yeosu, Jindo, and Jeju Island. Busan Station is located adjacent to Busan Port, a major trading port, where Argentine ants were detected in the cargo handling area. Since 2010, Busan Port has handled the largest volume of overseas shipping cargo in South Korea, with a total of 400 million tons of cargo processed in 2024 alone (National Logistics Information Center 2025). As international cargo transportation increases, so does the risk of unintentional introductions of alien species (Suarez et al. 2001; Hulme 2021), and the number of such introductions into South Korea has been steadily rising (Kim 2018; Park et al. 2016).

Ulsan, which was also identified as a high‐risk area, hosts Ulsan Port, the third‐largest port in South Korea in terms of overseas cargo volume. Previous detections of invasive alien species, such as *Niphe elongata* (Dallas, 1851) (Hemiptera: Pentatomidae) (Kim et al. 2025) and *Melanoplus differentialis* (Thomas, 1865) (Orthoptera: Acrididae) (Kang et al. 2022), indicate that major industrial and logistics hubs are particularly vulnerable to biological invasions. These patterns suggest that predicted high‐suitability areas associated with major ports and logistics centers should be a focus for monitoring and early detection of *L. humile* .

Jeju Island also faces a substantial risk of introduction and establishment as a major tourist destination with frequent movement of people and goods. While Yeosu and Jindo were predicted to have a high habitat suitability comparable to that of Busan, these regions currently lack major international ports or large floating populations. Therefore, the likelihood of introduction may be lower at present, but could increase if external logistics activities expand in the future.

### 4.5 Limitations and Future Research Directions

Despite the advantages of the ensemble approach, several limitations remain. Although occurrence data collection and model training have been conducted only in the Americas, sampling bias still exists, as it is impossible to record the occurrence of Argentine ants in all parts of the Americas at similar times and with the same effort and methods.

This model predicts species distribution based on information about the climate of the organisms. While climate significantly influences the distribution of organisms, it is not the only factor that determines their distribution. Some organisms may be tolerant of different climates, or interactions with other organisms may be more important for their distribution. Ants are similarly influenced by such nonclimatic factors, and collecting or creating environmental variables that reflect this is challenging.

Furthermore, ensemble modeling does not always yield better results than single models. Recent studies show that an ensemble model using default settings in BIOMOD ensemble platforms often results in performance similar to single models but does not show better performance. In some cases, appropriately tuned single models can perform as well as or even better than ensemble models (Hao et al. 2020). In another study comparing many different SDM variations, researchers concluded that there is no single best method for all cases. The authors recommend building several strong models and either selecting the best one through cross‐validation or using an average of their output instead of simply assuming that ensembles are always better (Norberg et al. 2019).

In this study, the chi‐square test was used as a complementary, distribution‐level assessment to evaluate whether the spatial pattern of suitable areas predicted by the ensemble model projections captured the observed occurrence data better than would be expected under a random distribution (Roura‐Pascual et al. 2004; Peterson et al. 2003). This approach assumes that the occurrence records used for testing are independent and that the ensemble model projections can be represented as categorical outcomes (presence/absence). However, spatial predictions derived from species distribution models are inherently spatially autocorrelated, and adjacent grid cells cannot be considered strictly independent. Furthermore, because the chi‐square test is based on binary classifications of predicted suitability, it does not incorporate information on the magnitude of predicted habitat suitability or the spatial structure of the predictions.

Future research should focus on a precise survey of Argentine ants across the entire Korean Peninsula. The new presence and absence data from this survey will be used as a test set to evaluate the current model. Comparing the predictions of this study with actual distribution data is important for the evaluation and adjustment of the model. Furthermore, it will be important to identify variables other than climate that influence species distribution and incorporate them into modeling, thereby exploring species distribution from a wider range of perspectives.

## Author Contributions

**Hyeban Namgung:** conceptualization (lead), data curation (lead), formal analysis (lead), investigation (lead), methodology (lead), project administration (supporting), software (lead), validation (lead), visualization (lead), writing – original draft (lead), writing – review and editing (equal). **Hyojoong Kim:** funding acquisition (lead), project administration (lead), resources (lead), supervision (lead), validation (supporting), writing – review and editing (equal).

## Conflicts of Interest

The authors declare no conflicts of interest.

## Supporting information


**Table S1:** List of environmental variables considered in modeling.
**Figure S1:** Points of 10 sets of *Linepithema humile* occurrence and pseudo‐absence data used for modeling in the Americas.
**Figure S2:** Points of occurrence data for *L. humile* outside the Americas used in the chi‐square test.
**Figure S3:** Results of multivariate environmental similarity surfaces analysis for the global projection of the ensemble model. (A) Variables combination set 1 (Bio03, Bio16, Bio18, and Soildegree); (B) variables combination set 2 (Bio04, Bio16, Bio18, and Soildegree); (C) variables combination set 3 (Bio04, Bio13, Bio18, and Soildegree).
**Figure S4:** Spatial distribution of suitable areas based on the mean annual cumulative soil temperature degree‐days above 15.9°C (Soildegree) (500–2000) in South Korea.
**Figure S5:** Spatial distribution of suitable areas based on the temperature seasonality (Bio04) (500–800) in South Korea.

## Data Availability

The data that support the findings of this study are openly available in Dryad at https://doi.org/10.5061/dryad.x0k6djhz3.
