# Supplementary material for: Potential Distribution of Linepithema humile (Hymenoptera: Formicidae) in South Korea: An Ensemble Species Distribution Modeling Approach
Source: Ecol Evol. 2026 Feb 3;16(2):e72976. doi: 10.1002/ece3.72976 (PMC12865865; doi:10.1002/ece3.72976)
Supplement: Supplementary file 1 — Table S1: List of environmental variables considered in modeling. Figure S1: Points of 10 sets of Linepithema humile occurrence and pseudo‐absence data used for modeling in the Americas. Figure S2: Points of occurrence data for L. humile outside the Americas used in the chi‐square test. Figure S3: Results of multivariate environmental similarity surfaces analysis for the global projection of the ensemble model. (A) Variables combination set 1 (Bio03, Bio16, Bio18, and Soildegree); (B) variables combination set 2 (Bio04, Bio16, Bio18, and Soildegree); (C) variables combination set 3 (Bio04, Bio13, Bio18, and Soildegree). Figure S4: Spatial distribution of suitable areas based on the mean annual cumulative soil temperature degree‐days above 15.9°C (Soildegree) (500–2000) in South Korea. Figure S5: Spatial distribution of suitable areas based on the temperature seasonality (Bio04) (500–800) in South Korea. [file ECE3-16-e72976-s001.docx]

| **Environmental variables** | **Maximum** | **Minimum** | **Mean** | | **SD** |
| --- | --- | --- | --- | --- | --- |
| **Annual mean temperature (Bio01)** | 29.52 | -11.96 | 17.23 | 8.05 | |
| **Mean diurnal range (Bio02)**  **(Mean of monthly (max temp - min temp))** | 21.82 | 3.77 | 12.19 | 2.61 | |
| **Isothermality (Bio03)**  **(BIO02/BIO07 × 100)** | 94.83 | 2.08 | 56.17 | 18.9 | |
| **Temperature seasonality (Bio04)**  **(Standard deviation ×** **100)** | 1371.82 | 8.52 | 458.2 | 380.8 | |
| **Max temperature of warmest month (Bio05)** | 46.03 | -2.3 | 29.92 | 4.93 | |
| **Min temperature of coldest month (Bio06)** | 24.2 | -2.7 | 4.73 | 12.78 | |
| **Temperature annual range (Bio07)**  **(BIO05-BIO06)** | 51.4 | 7.22 | 25.19 | 11.35 | |
| **Mean temperature of wettest quarter (Bio08)** | 33.59 | -1.52 | 19.67 | 7.84 | |
| **Mean temperature of driest quarter (Bio09)** | 31.08 | -1.73 | 14.51 | 11.51 | |
| **Mean temperature of warmest quarter (Bio10)** | 36.15 | -7.66 | 22.7 | 5.15 | |
| **Mean temperature of coldest quarter (Bio11)** | 28.28 | -1.73 | 11.47 | 12.2 | |
| **Annual precipitation (Bio12)** | 7297 | 0 | 1211.75 | 810.08 | |
| **Precipitation of wettest month (Bio13)** | 927 | 0 | 180.18 | 116.63 | |
| **Precipitation of driest month (Bio14)** | 498 | 0 | 37.17 | 41.88 | |
| **Precipitation seasonality (Bio15)**  **(Coefficient of Variation)** | 200.78 | 0 | 53.1 | 27.65 | |
| **Precipitation of wettest quarter (Bio16)** | 2461 | 0 | 492.19 | 323.29 | |
| **Precipitation of direst quarter (Bio17)** | 1547 | 0 | 131.45 | 137.28 | |
| **Precipitation of warmest quarter (Bio18)** | 1876 | 0 | 304.73 | 193.1 | |
| **Precipitation of coldest quarter (Bio19)** | 2281 | 0 | 286.7 | 278.62 | |
| **mean annual cumulative degree‑days above 15.9 °C** | 5985.39 | 0 | 2148.26 | 1466.3 | |

**Table S1 List of environmental variables considered in modeling.**


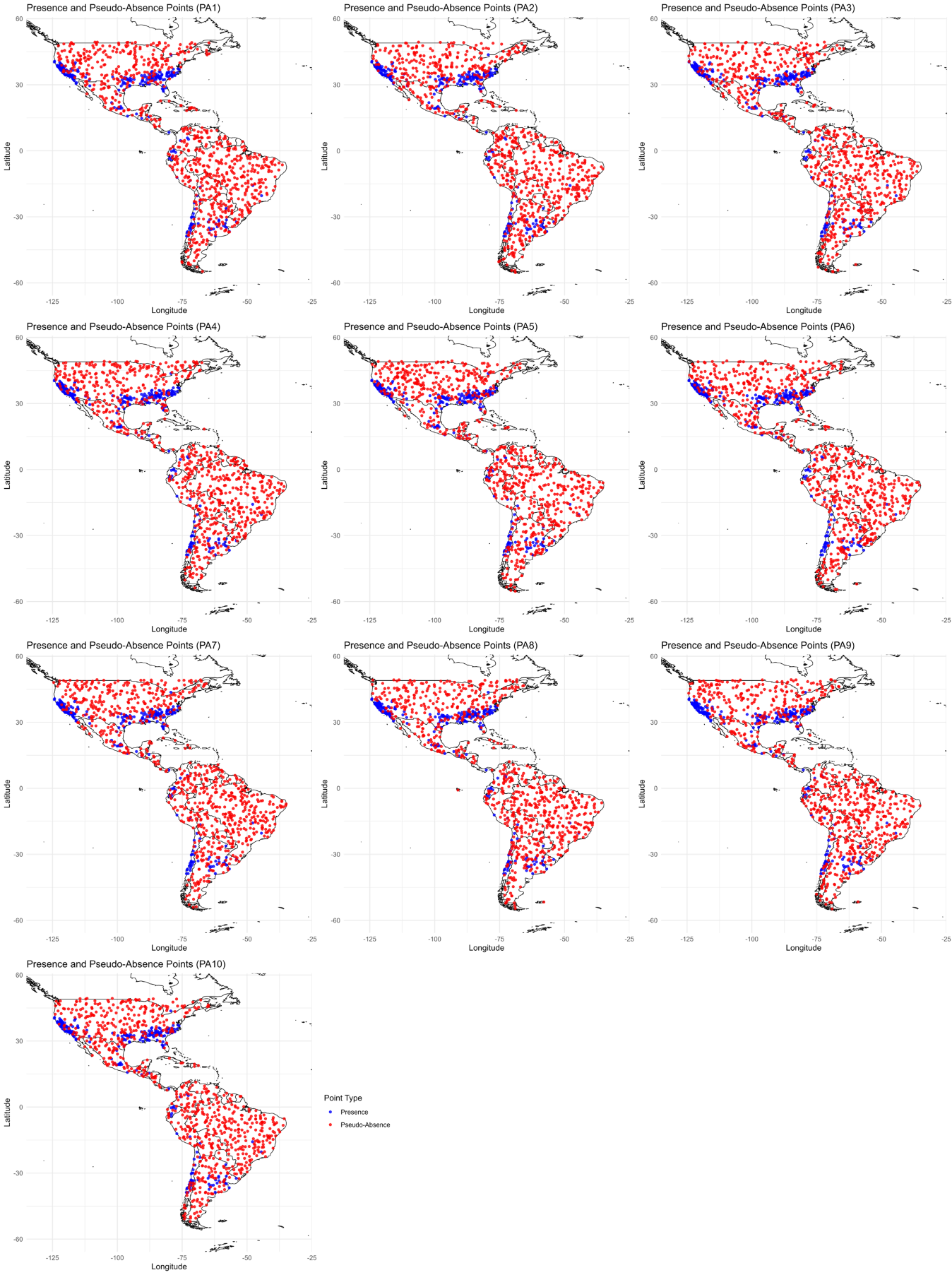


Figure S1 Points of ten sets of *Linepithema humile* occurrence and pseudo-absence data used for modeling in the Americas.


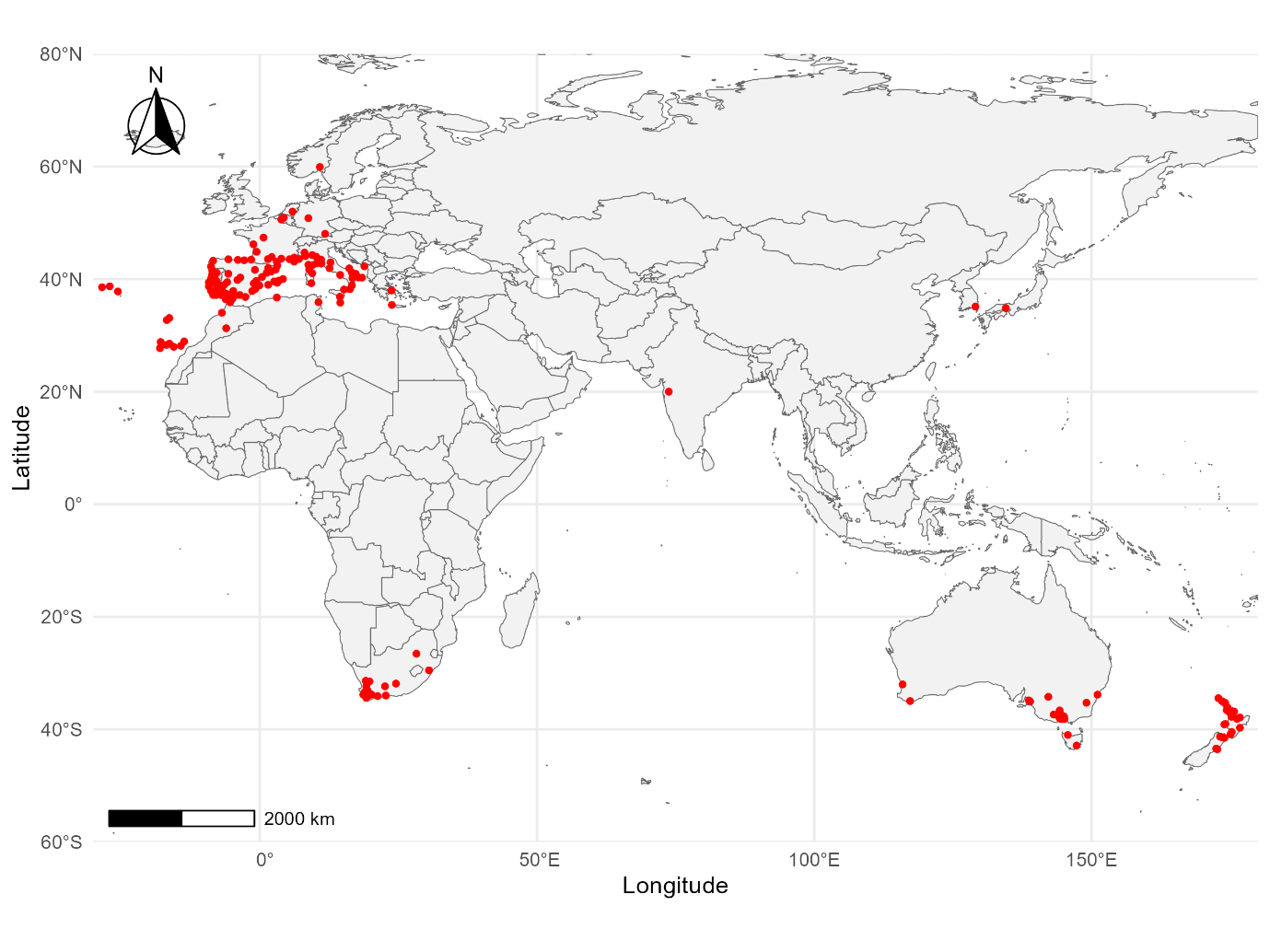
Figure S2 Points of occurrence data for *L. humile* outside the Americas used in the chi-square test.


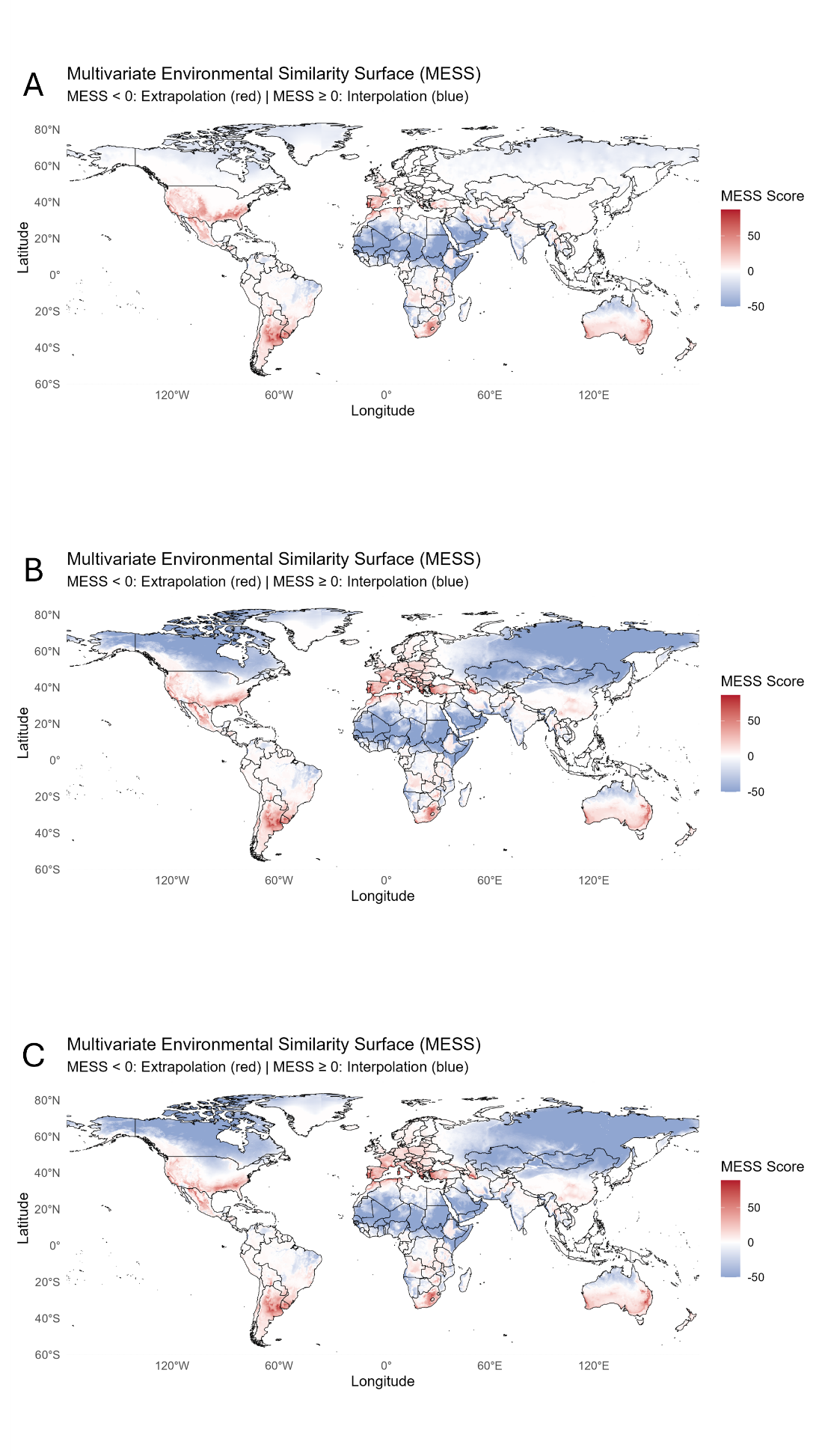


Figure S3 Results of multivariate environmental similarity surfaces analysis for the global projection of the ensemble model. A, variables combination set 1 (Bio03, Bio16, Bio18, and Soildegree); B, variables combination set 2 (Bio04, Bio16, Bio18, and Soildegree); C, variables combination set 3 (Bio04, Bio13, Bio18, and Soildegree).


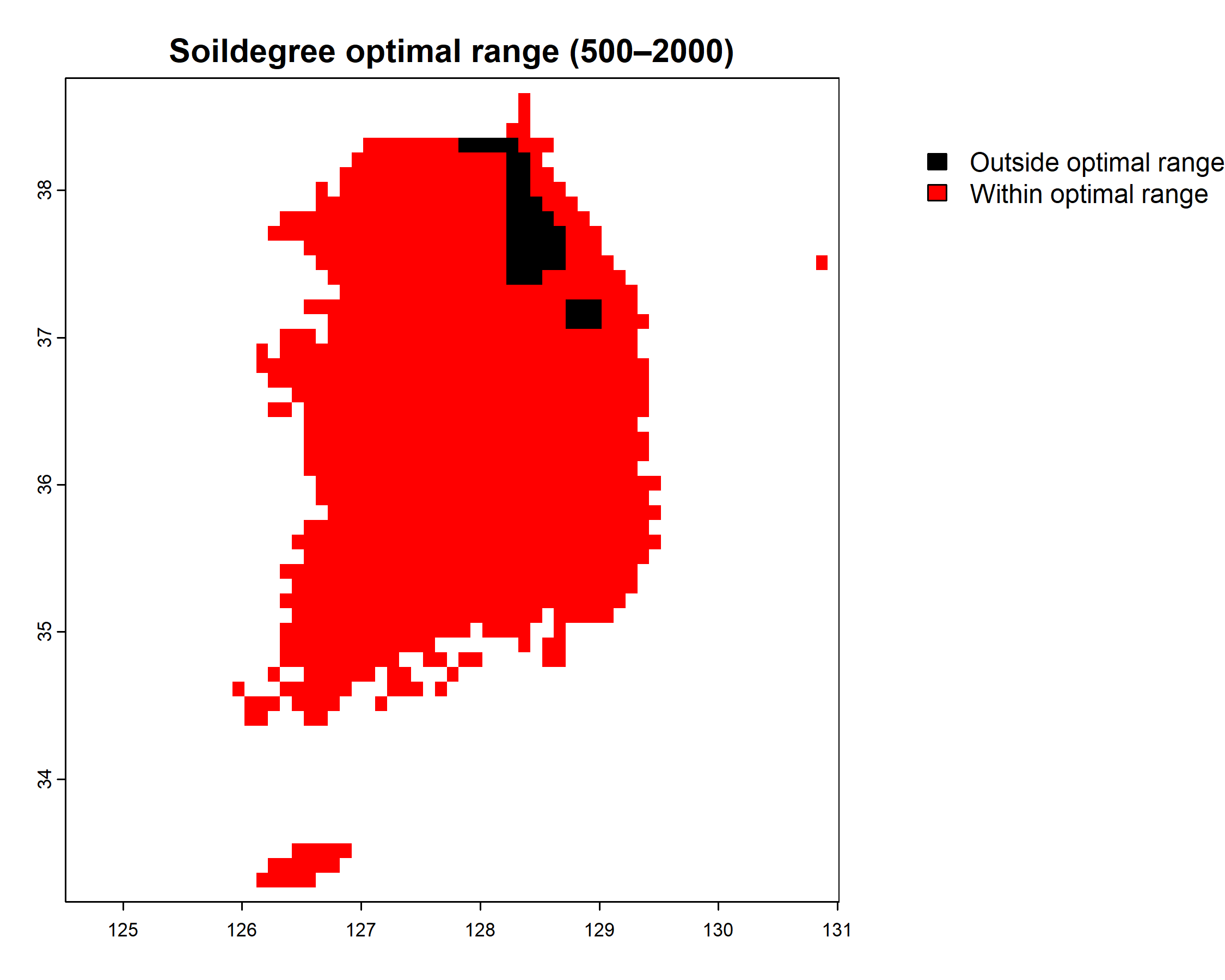


Figure S4 Spatial distribution of suitable areas based on the mean annual cumulative soil temperature degree‑days above 15.9 ℃ (Soildegree) (500–2000) in South Korea


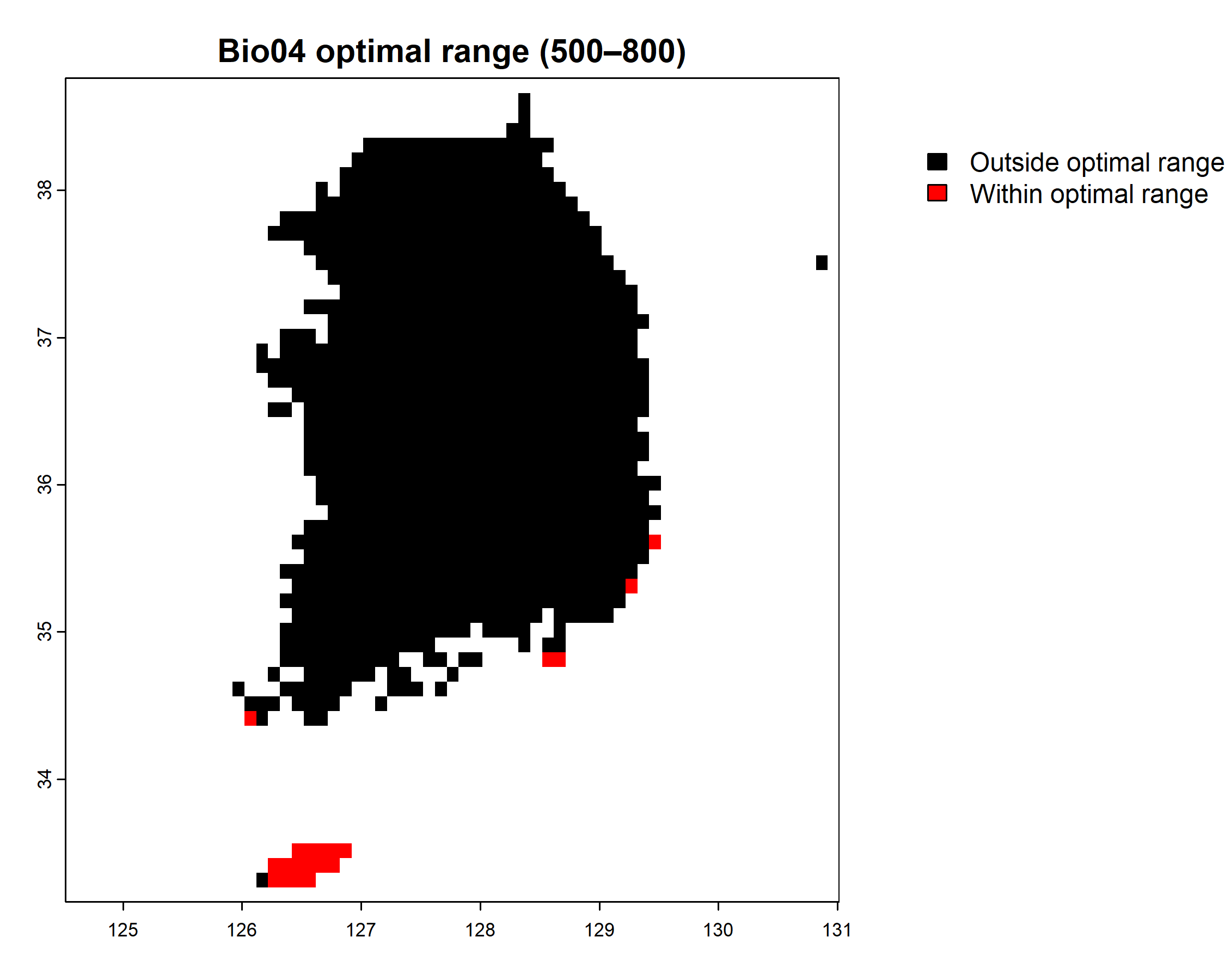


Figure S5 Spatial distribution of suitable areas based on the temperature seasonality (Bio04) (500–800) in South Korea
